# Energy loss function of samarium

**DOI:** 10.1038/s41598-023-30770-1

**Published:** 2023-03-08

**Authors:** T. F. Yang, R. G. Zeng, L. H. Yang, A. Sulyok, M. Menyhárd, K. Tőkési, Z. J. Ding

**Affiliations:** 1 Department of Physics, University of Science and Technology of China, Hefei, 230026 Anhui People’s Republic of China; 2 Hefei National Research Center for Physical Sciences at the Microscale, University of Science and Technology of China, Hefei, 230026 Anhui People’s Republic of China; 3 Institute of Materials, China Academy of Engineering Physics, P.O. Box 9071, Jiangyou, 621907 Sichuan People’s Republic of China; 4 Centre for Energy Research, Research Institute for Technical Physics and Materials Science, ELKH, P.O. Box 49, H-1525 Budapest, Hungary; 5 Institute for Nuclear Research, ELKH, P.O. Box 51, Debrecen, Hungary

**Keywords:** Materials science, Physics

## Abstract

We present a combined experimental and theoretical work to obtain the energy loss function (ELF) or the excitation spectrum of samarium in the energy loss range between 3 and 200 eV. At low loss energies, the plasmon excitation is clearly identified and the surface and bulk contributions are distinguished. For the precise analysis the frequency-dependent energy loss function and the related optical constants (*n* and *k*) of samarium were extracted from the measured reflection electron energy loss spectroscopy (REELS) spectra by the reverse Monte Carlo method. The *ps*- and *f*-sum rules with final ELF fulfils the nominal values with 0.2% and 2.5% accuracy, respectively. It was found that a bulk mode locates at 14.2 eV with the peak width ~6 eV and the corresponding broaden surface plasmon mode locates at energies of 5-11 eV.

## Introduction

Lanthanides have nowadays become of vital importance in advanced materials and technology. Applications in laser science^1,2^, solar cells^3^, fluorescent lamps and a new organic light-emitting diodes components^4–6^, as well as luminescent probes^7^ are strongly related with their optical and/or electronic properties. Samarium^8–11^ and its compounds^12–18^ are among the most frequently used lanthanides in the investigations during the last years. But the precise excitation property, especially the plasmon structure of samarium is still not known. It is not surprising because all lanthanides are highly reactive elements and interact strongly with oxygen and hydrogen. The excitation properties are intrinsically embodied in the energy loss function (ELF), ${\text{Im}}\left\{ {{{ - 1} \mathord{\left/ {\vphantom {{ - 1} {\varepsilon \left( \omega \right)}}} \right. \kern-0pt} {\varepsilon \left( \omega \right)}}} \right\}$, which is clearly related with the frequency dependent complex dielectric function $\varepsilon \left( \omega \right)$, or equivalently in the refractive index $n\left( \omega \right)$ and the extinction coefficient $k\left( \omega \right)$. Therefore, the accurate knowledge of these functions is crucial partly for identifying various excitations and also for the new applications. The optical measurements (by reflectance and absorbance), however, are usually performed under atmospheric conditions and this should be a reason why there are only a few measurements for optical constants, $n\left( \omega \right)$ and $k\left( \omega \right)$, available in literature for Sm^19^. Knyazev and Noskov had reviewed the study of the optical properties of the lanthanides^20^, where the optical conductivity was provided in the low photon energy region (below 5 eV) by optical reflection or absorption measurements. At higher photon energies (above 70 eV), reliable optical data can be deduced from X-ray-solid interaction experiments^21–23^. There are also a few data available in the intermediate photon energy region, around several tens eV, where the rich structure of the transition lines from 4f electrons and the 6s and 5p electrons of samarium are located. Netzer et al.^24^ had performed a comprehensive study of reflected electron energy loss spectroscopy (REELS) experiments to probe valence excitations (0–50 eV) for all the lanthanides. Although several features were resolved using the REELS spectra at various primary energies, the characteristic transitions behind these peaks were not identified. For example, for the sharp peak at ~3 eV in the REELS spectra of Sm, the authors followed a previous interband transition explanation by Bertel et al.^25^ based on an indirect electron energy loss spectroscopy (EELS) observation for Sm^26^. Yubero and Tougaard^27^ developed a method (YT-model) to derive the DIIMFP and the ELF from a REELS spectrum, where the trial-and-error procedure is employed to determine the best fit ELF. This method had been successfully applied to the research of the optical constants of Fe, Pd and Ti^28^.

Since the characteristic structures of a REELS spectrum, especially in the intermediate energy loss region, inevitably involve combined effects from plasmon excitations, interband transitions and the multiple inelastic collisions, it is important and preferable analyzing critically the optical constants or dielectric response function to identify the energy loss peak structure of Sm. In this work, we present a combined experimental and theoretical investigation of the ELF or the excitation spectrum of samarium in the energy loss range between 3 and 200 eV. The plasmon excitation is clearly identified and the surface and bulk contributions are distinguished at low energy part of the excitation spectrum. Furthermore, the excitation spectrum up to 200 eV is determined. For the precise analysis, firstly, the reverse Monte Carlo (RMC) method^29–38^ was used to extract ELF and, thereby, the optical constants and relevant electronic properties from the measured REELS spectra. The RMC method combines a Monte Carlo modelling of electron transportation and REELS spectrum simulation with a Markov chain Monte Carlo calculation of the ELF. The REELS measurement was carried out in an UHV condition to obtain data of pure material. Compared with optical methods, the energy loss spectrum was measured in a rather wide energy loss range of 3–200 eV, which enables to obtain optical constants in the corresponding photon energy range by a single measurement. In contrast with the transmission mode of EELS, the experimental measurement with the reflection mode of EELS has advantages: (a) the typical incident energy of primary electrons can be much lower in the order of keV range; (b) in addition, there is no special requirement for sample preparation. At the same time, however, applying lower primary energy, the surface excitation effect becomes important in a REELS measurement. Therefore, in the RMC method, the elastic scattering, the surface- and bulk-excitations and the multiple scattering effects are taken into account in order to extract the pure bulk optical property of material.

## Dielectric function

Based on the free-electron model^39^ which assumes that the target medium has a homogeneous electron density, the Drude type ELF is given by

1 $${\text{Im}} \left[ {\frac{ - 1}{{\varepsilon_{D} \left( {\omega ;\omega_{p} ,\gamma } \right)}}} \right] = \frac{{\omega \gamma \omega_{p}^{2} }}{{\left( {\omega^{2} - \omega_{p}^{2} } \right)^{2} + \omega^{2} \gamma^{2} }},$$

where $\omega_{p} = \sqrt {{{4\pi ne^{2} } \mathord{\left/ {\vphantom {{4\pi ne^{2} } m}} \right. \kern-0pt} m}}$ is plasmon frequency, *n* is the electron density, and $\gamma$ denotes the full width at half maximum of the plasmon peak. In the RMC simulation, a bulk ELF for an unknown material, which is a non-free-electron-gas metal, is represented by a finite sum of Drude terms^40,41^,

2 $${\text{Im}} \left[ {\frac{ - 1}{{\varepsilon \left( \omega \right)}}} \right] = \sum\limits_{{\text{i = 1}}}^{{\text{N}}} {A_{i} {\text{Im}} \left[ {\frac{ - 1}{{\varepsilon_{D} \left( {\omega ,\omega_{pi} ,\gamma_{i} } \right)}}} \right]},$$

and Ritchie and Howie’s scheme^41^ is employed to extend the dielectric function from the optical limit to other momentum transfers,

3 $${\text{Im}} \left[ {\frac{ - 1}{{\varepsilon \left( {q,\omega } \right)}}} \right] = \sum\limits_{{\text{i = 1}}}^{{\text{N}}} {A_{i} {\text{Im}} \left[ {\frac{ - 1}{{\varepsilon_{D} \left( {q,\omega ;\omega_{pi} ,\gamma_{i} } \right)}}} \right]}.$$

Drude-type dielectric function is given by

4 $$\varepsilon_{D} \left( {q,\omega ;\omega_{pi} ,\gamma_{i} } \right) = 1 + \frac{{\omega_{pi}^{2} }}{{\omega_{qi}^{2} \left( {q,\omega_{pi} } \right) - \omega_{pi}^{2} - \omega \left( {\omega + i\gamma_{i} } \right)}}.$$

For the extension of optical ELF, the following dispersion relation is employed,

5 $$\omega_{qi}^{2} \left( {q,\omega_{pi} } \right) = \omega_{pi}^{2} + 2E_{F} {{q^{2} } \mathord{\left/ {\vphantom {{q^{2} } 3}} \right. \kern-0pt} 3} + {{q^{4} } \mathord{\left/ {\vphantom {{q^{4} } 4}} \right. \kern-0pt} 4}.$$

The present REELS-RMC method embedded in our CTMC-RMC code^42^ is based on the physical description of electron-bulk and electron-surface interactions without any artificial scaling factor in our model. In this way, the method derives the absolute values of ELF while the validity of the data can be checked by the sum rules^36,38^.

## Experiment

**Figure 1:**
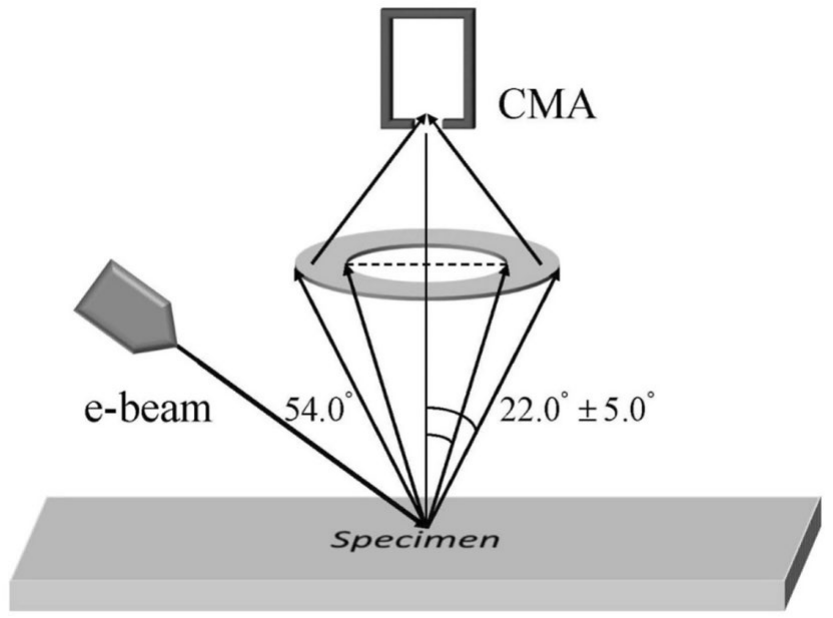
Schematic diagram of the REELS experimental configuration.

The samarium sample was a pellet which exhibited rather rough oxidized surface. Therefore, the sample was first mechanically polished by alumina powder (made by Buehler, 1 µm grain size) in water suspension to remove the deep saw tracks from the surface. After water rinsing, and acetone washing, the pellet was immediately introduced into vacuum system. After 48 hours baking at 80 °C, the surface was ion bombarded by 1 keV Ar^+^ beam until reaching carbon free surface. Ion bombardment was repeated sequentially to maintain the clean surface during the whole measurement. The surface of the sample before and after the measurement of REELS spectra was checked by measuring the Auger peaks of Sm at 154 eV, C at 272 eV and O at 510 eV. Only measurements exhibiting C and O concentration lower than detectability level of 4% were accepted. The REELS spectrum was recorded in an energy range between 0 and 200 eV at 2 keV primary energy. The backscattered electrons were detected by a retarded cylindrical mirror analyzer (DESA 150, Staib Instruments Ltd.) applying a constant energy resolution of 2 eV. The incident angle of the primary electron beam was 54° with respect to the surface normal of the sample and the electrons emitted in the polar angle range of 22°±5° (with respect the surface normal of the specimen) were measured, as shown in Fig. 1.

## Results and discussions

Monte Carlo simulations of the REELS spectrum were performed with the same conditions as the experiments. The ELF or complex dielectric function is not related to the experimental condition and should not be dependent on the primary energy of the incident electron beam. In the following all analysis will be related to the primary energy of 2 keV, but to verify that the obtained ELF is independent of the primary energy, the measurements and simulations for 0.5 keV and 1 keV were also performed. A converged result is obtained by the RMC procedure, and an extremely good agreement between the simulated and the experimental REELS spectra are achieved in the whole energy loss range. This good agreement between theory and experiment also indicate that the obtained ELF is very accurate.

**Figure 2:**
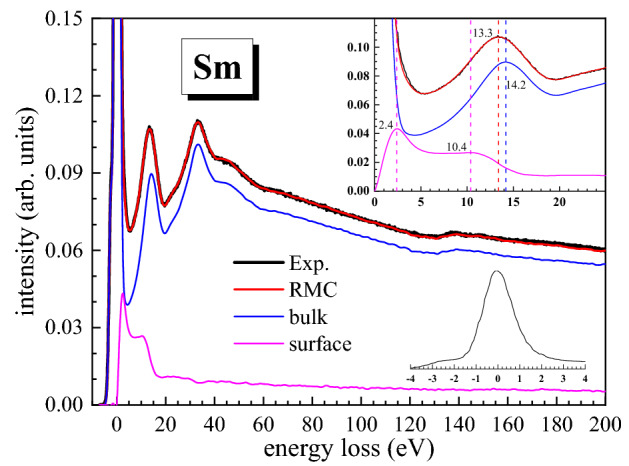
Comparison of the final simulated REELS spectrum (red) of Sm with experimental spectrum (black) for a primary electron beam of 2 keV. Pure bulk excitation component (blue) and surface excitation component (magenta) are also calculated based on the derived ELF by the Monte Carlo simulation. The upper inset shows the low loss region, and the lower inset illustrates the energy distribution of the elastic peak.

Fig. 2 shows the final simulated REELS spectrum of Sm with the experimental one. The simulation of the full REELS spectrum was performed with the differential inverse inelastic mean free path (DIIMFP) containing both bulk and surface excitations^29^. The contributions individually from the bulk and surface excitations are also shown in Fig. 2, where the bulk component is simulated with the DIIMFP containing only the bulk term^43^ and the surface component is a difference between the full spectrum and the bulk component. The simulated spectra are convoluted with Gaussian functions for the elastic peak width ~1.3 eV reflecting the original energy distribution of the hot cathode of the electron gun to mimic the experimental measured elastic peak shape. Both the simulated and experimental spectra are normalized at the zero-loss peak intensity. The simulated REELS spectrum will be mentioned hereafter as the full spectrum containing both the bulk and surface excitation components.

**Figure 3:**
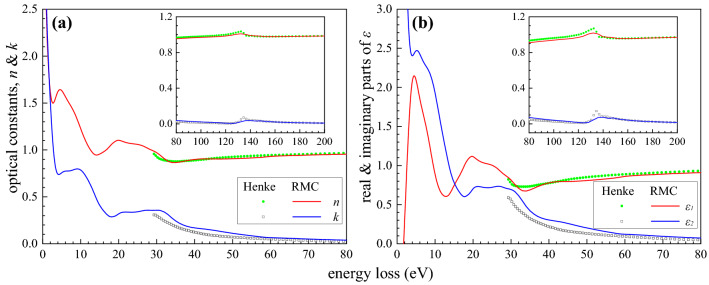
(**a**) A plot of the refractive index $n$ (red) and extinction coefficient $k$ (blue) of Sm derived from ELF by the RMC analysis. Optical experimental data^22^ (dots and squares for $n$ and $k$, respectively) are shown for a comparison; (**b**) A plot of real part $\varepsilon_{1}$ (red) and imaginary part $\varepsilon_{2}$ (blue) of the complex dielectric function of Sm derived by the RMC method. Data given in Ref.^22^ (dots and squares for $\varepsilon_{1}$ and $\varepsilon_{2}$, respectively) are shown for a comparison.

The optical constants can be derived from the complex dielectric function through the ELF via Kramers-Kronig relation. Fig. 3 shows the obtained refractive index $n$ and the extinction coefficient $k$ in comparison with the literature data^22^. As it can be seen in Fig. 2 that the shape of the full spectrum (and also the experimental spectrum) is quite similar to the bulk spectrum in the energy loss region above 15 eV, indicating that the bulk excitation dominates above 15 eV. A shift of 0.8 eV for the 14.2 eV peak in the bulk spectrum compared to the full spectrum displays that the surface excitation plays an important role in the low loss region. The information about very low energy losses below 3 eV is buried in the elastic peak of the experimental spectrum, and the obtained data may lose accuracy in this range. A plateau around 5-11 eV characterizes the surface excitation spectrum, and no other surface excitation features were observed in the higher loss region than 15 eV, indicating very weak multiple scattering effects in the surface region of the sample.

**Figure 4:**
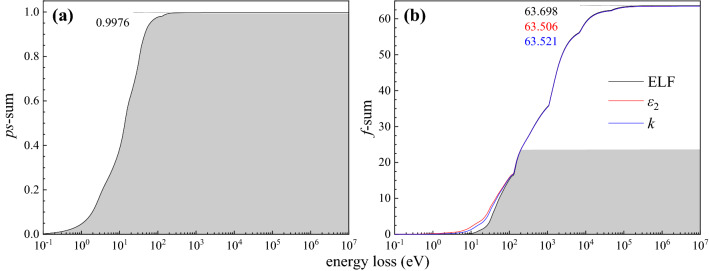
Calculation of *ps*-sum for ELF (**a**) and* f*-sum (**b**) rules for ELF (black), $\varepsilon_{2}$ (red) and *k* (blue) of Sm obtained from RMC method. Grey area indicates the contribution from the present data (0-200 eV), and the rest part is due to the data of Ref.^22^ (200 eV-30 keV) and the data of Ref.^44^ (30 keV-10 MeV).

The accuracy of the present ELF was checked by applying the perfect-screening (*ps*-sum) and the Thomas-Reiche-Kuhn (*f*-sum) sum rules^44^. The sum rules were calculated by using the present ELF, imaginary part of the complex dielectric function $\varepsilon_{2}$, or extinction coefficient $k$ data below 200 eV in combination with data from Ref.^22^ (200 Ev–0 keV) and Ref.^45^ (30 keV–10 MeV) (see Fig. 4). The theoretical values for *ps*- and *f-*sum rules are, respectively, 1 and the atomic number (i.e. 62 for Sm). The grey area in Fig. 4 highlights the contributions from the present ELF, $\varepsilon_{2}$, or $k$. It is 99.6% for *ps*-sum rule and about 37.0% for *f*-sum rules. In Fig. 4(a), the convergence of the *ps*-sum rule is reached at 0.9976, which is very close to the nominal theoretical value of unity. For the current integration limit and for the current supplementary data, the results of *f*-sum rules of Sm for ELF, $\varepsilon_{2}$, and $k$ are 63.698, 63.506, and 63.521, respectively, resulting in a small averaged relative error of 2.5 % to the theoretical value. Therefore, the high accuracy of the recent results obtained by RMC analysis is confirmed by both the *ps*- and *f*-sum rules. It is worth noting that according to the weighting factors, *ω*^-1^ for the case of *ps*-sum rule mainly emphasizes the low energy region, and *ω* for the case of *f*-sum rule is more affected in the high energy region. Therefore, the obtained 2.5 % relative error for the *f*-sum rule most likely can be attributed to the data above 200 eV.

**Figure 5:**
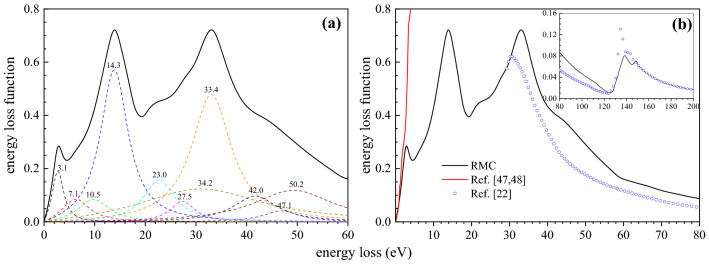
(**a**) A plot of ELF deduced from the experimental REELS spectrum by RMC method (solid line) together with 11 Drude terms (dashed lines); (**b**) Comparison on ELF of Sm between the present RMC result (black), Ref.^22^ (blue circles) and Ref.^47,48^(red line).

The Markov chain Monte Carlo process, whose principle is based on a search for the global minimum on a potential energy surface in a multiparameter space, is used in the RMC method to find the optimal ELF; the simulated annealing method^46^ is employed to achieve our purpose. Fig. 5(a) shows the bulk ELF together with the fitted first 11 Drude functions characterizing completely the energy loss region below 60 eV. The parameters of the Drude terms are summarized in Table 1. Fig. 5(b) shows the comparison between present ELF result and data measured by Knyazev and Noskov^47,48^ in the low loss region. Although an agreement below 1 eV between the two ELFs can be found, the Knyazev’s data increases drastically in the range of 2.0-3.9 eV, whose behavior seems to be quite abnormal. It shows also the ELF derived from the atomic scattering factors by an X-ray-samarium interaction measurement in the higher energy loss region (above 29 eV)^22^. Since the atomic scattering factor describes the properties of a single atom, while the ELF of solids in the low energy loss region is determined by its valence electrons, there is a discrepancy between the present data and Henke's data in the energy loss region below 150 eV. Generally, the decreasing trend with increasing energy loss is found in both datasets and especially above 150 eV they have achieved a good agreement.

**Table 1:** Parameters used to determine bulk ELF of Eq. (2) in the energy loss range up to 60 eV.

*A*_*i*_	*ω*_*pi*_/eV	*γ*_*i*_/eV
0.168	3.1	2.9
0.079	7.1	6.9
0.060	10.5	7.8
0.264	14.3	6.7
0.051	23.0	7.5
0.016	27.5	5.9
0.136	33.4	9.6
0.104	34.2	30.1
0.024	42.0	10.6
0.008	47.1	8.7
0.045	50.2	19.1

The excitation characteristics from the present ELF are richer than those observed at ~2.6, 7.5 and 12 eV in the REELS spectra in Ref.^25^ or at ~3 eV in Ref.^24^. The transitions behind these lines are not well defined via merely EELS experiments. The distribution of the signal electrons collected in a REELS experiment always contains the effect of multiple scattering effect, especially in the reflection mode. In addition, the zero-loss peak width and resolution of the detector will increase the difficulty of the analysis of peaks, especially for low energy loss region. The characteristic features in the spectra sometimes show a shifting behavior at different primary energies^24,25^ because of varying contributions from surface and bulk contributions as already discussed for the shift of the 14.2 eV peak of the bulk spectrum in Fig. 2. These intrinsic properties are therefore more precisely accessed via the fundamental physical parameters, namely, the dielectric function.

Only a few previous works have reported the plasmon excitation of samarium. Dufour et al. used X-ray photoelectron spectroscopy (XPS) to study REELS spectra of 4 keV electrons reflected from Sm and Sm_2_O_3_ samples together with the XPS spectra of emitted photoelectrons; a peak around 11.5 eV was found^49^. The peak near 12 eV in REELS spectra was interpreted as a bulk plasmon whose theoretical value is 11.2 eV with a hypothesis of three free electrons per atom in Ref.^25^. Netzer used a free electron model to obtain a predictive bulk plasmon energy of ~11.8 eV and reported a 13.5 eV peak from REELS spectra^24^. However, according to the present ELF from the RMC analysis the sharp peak at 14.3 eV in Fig. 5(a) is identified as an actual bulk plasmon. The 13.3 eV peak (near to the reported 12 eV peak) in REELS spectrum in Fig. 2 can be explained as mixed contributions from of a surface plasmon excitation at 10.4 eV and a bulk plasmon excitation at 14.2 eV; this peak shifting is identified by the simulation, that is, the first strong peak ~13.3 eV in the measured REELS spectrum has been well separated into the bulk and the surface spectra.

In addition, in the higher energy loss region, a small hump was identified at 27.5 eV in Fig. 5(a), which are close to the peak with the energy of 27.0 eV from the atomic experimental works^50^. This feature can be interpreted as an atomic (5p; 5d) ^1^P resonance of the type suggested by Wendin^51^. This atomic resonance exhibits a strong absorption above the 5p_3/2_ ionization edge^50^. A wide shoulder covering the 19-25 eV region and a strong asymmetric 33.4 eV peak are observed also in Fig. 5(a), indicating the complex composition of transitions. These two structures were investigated as (5p; 5d) ^1^P resonance of solid at 22.5 eV and 33.0 eV in Ref.^52^ via observation of EELs spectra carried out at different primary energies. In addition to these features, possible transitions covering the extreme ultraviolet (EUV) region (<60 eV) are also presented in Fig. 5(a).

**Figure 6:**
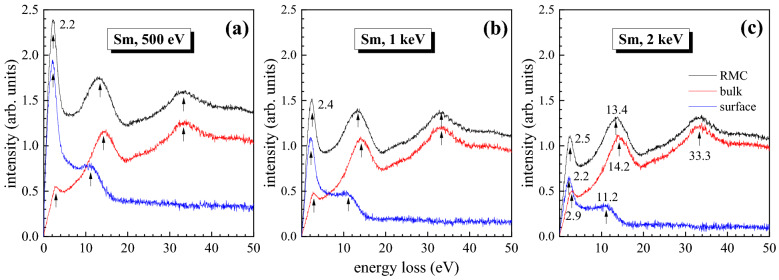
Comparison between the simulated REELS spectrum (black), pure bulk excitation component (red) and surface excitation component (blue), in which the peak broadening due to the elastic peak distribution has been removed.

The sharp 2.6 eV peak in Refs.^24,25,53^ has been interpreted as an interband transition. The final result also shows a peak at 3.1 eV in ELF (Fig. 5(a)); however, the detailed information about 2.4 eV peak in Fig. 2 is hidden because of the elastic peak width. Since an ideal Gaussian distribution for the elastic peak was used, any distortion of the elastic peak shape is attributed to the low energy losses and this approximation may lose accuracy for the analysis of peak features below 3 eV. Fig. 6 shows the simulated REELS spectra with pure bulk and surface excitation components at 500 eV, 1 keV and 2 keV incident energies. The surface plasmon mode has broaden energies below 11 eV. The surface plasmon excitation will play a much more important role in the low primary energy as it is expected that the surface plasmon excitation becomes more and more significant and dominant with decreasing primary energy. This feature of the energy dependence also gives a clear indication that the calculated surface mode spectrum comes indeed from the surface plasmon excitation. The peak near 7.5 eV (7.1 eV in the Fig. 5(a)) for Sm is determined as an one-electron excitation rather than the surface plasmon in Ref.^25^, in exact agreement with the energy position of the peak connected with the excitation of 4f electrons in an X-ray photoemission investigation^20,54^. Such transition was also investigated in Ref.^55^, where this peak is split into two peaks at 5.8 eV and 7.5 eV. The peak splitting can also be observed for the extinction coefficient in Fig. 3(a).

In conclusion, the excitation energies of samarium in the energy range between 3 eV and 200 eV were studied. First the RMC technique was used to obtain the electron energy loss features buried in the REELS spectra of samarium. The accuracy of the ELF was checked by applying the Thomas-Ritchie-Kuhn (*f*-sum rule) and the perfect-screening sum rules (*ps*-sum rule). The *ps*- and *f*-sum rules with the present ELF, imaginary part of the complex dielectric function $\varepsilon_{2}$, and extinction coefficient $k$ fulfils very accurately and reach the nominal values with 0.2% and 2.5% accuracy, respectively. The contribution from the bulk and the surface excitations has been shown. Furthermore, the detailed excitation characteristic in the optical data in the energy range between 3 and 60 eV were identified and the structures were clearly assigned to the corresponding processes. A surface plasmon mode was found at 10.4 eV, and the corresponding bulk mode at 14.3 eV. Moreover, the excitation lines up to 200 eV are also shown and determined.

## Data Availability

The datasets generated and/or analyzed during the current study are available from the corresponding authors on reasonable request.
